# Supplementary material for: WordCommentsAnalyzer: A windows software tool for qualitative research
Source: F1000Res. 2018 Sep 4;7:536. Originally published 2018 May 3. [Version 2] doi: 10.12688/f1000research.14819.2 (PMC6137411; doi:10.12688/f1000research.14819.2)
Supplement: Supplementary file 3 [file f1000research-7-17614-s0002.tgz › b2a79496-4ada-47c4-9682-a99ce867836d.zip!/Eight Iranian Health Journals/Yafteh _8.docx]

© Yafteh  . This work is licensed under CC BY 4.0 (https://creativecommons.org/licenses/by/4.0/).

Volume & Issue: دوره 18 - شماره 2

Title: بررسی میزان آگاهی، نگرش و عملکرد مادران دارای کودکان 36-6 ماهه تحت پوشش مراکز بهداشت شهر خرم آباد در مورد نحوه تغذیه صحیح کودکان خود در سال 94

http://yafte.lums.ac.ir/article-1-2291-fa.html

مقدمه: مهم‌ترین عوامل تأثیر گذار بر وضعیت تغذیه‌­ای کودک و بهبود آن کودک آگاهی، نگرش و عملکرد مادر در مورد تغذیه صحیح کودک خود است. این پژوهش به‌منظور سنجش میزان آگاهی، نگرش و عملکرد مادران دارای کودکان 36-6 ماهه تحت پوشش مراکز بهداشت شهر خرم آباد در مورد نحوه تغذیه صحیح کودکان خود انجام گرفت. مواد و روش ­ها: در این مطالعه مقطعی توصیفی-تحلیلی اطلاعات مربوط به 302 مادر دارای کودک 36-6 ماهه مراجعه کننده  به مراکز بهداشتی درمانی شهر خرم آباد از طریق پرونده‌های بهداشتی مادران و روش مصاحبه جمع آوری گردید و داده‌ها با استفاده از نرم افزار SPSS21 و آزمون استقلال مجذور کای و ضریب همبستگی اسپیرمن و پیرسن مورد بررسی قرار گرفتند. یافته ­ها: بر اساس یافته‌ها و امتیازات کسب شده توسط مادران 9/69% زنان دارای آگاهی خوب 9/69% دارای نگرش خوب و 6/64% دارای عملکرد خوب در مورد تغذیه صحیح کودکان خود بودند. بین سطح نگرش و تحصیلات مادر، تحصیلات همسر، شغل مادر ارتباط معنی دار وجود داشت. بین سطح عملکرد و تحصیلات همسر، شغل همسر، تعداد فرزند، رتبه تولد ارتباط معنی دار وجود داشت. رابطه معنی داری بین میزان توجه به رسانه‌های گروهی با نمره آگاهی و نگرش وجود نداشت اما با عملکرد ارتباط معناداری داشت. بین سطح آگاهی و تحصیلات مادر، تحصیلات همسر، شغل مادر، شغل همسر، درآمد، تعداد فرزند و رتبه تولد ارتباط معنی دار وجود نداشت. بحث و نتیجه­ گیری: با توجه به نتایج مطالعه مذکور، میزان آگاهی، نگرش و عملکرد مادران در سطح خوبی است ولی با توجه به نقش بالقوه پدر خانواده در عملکرد مادر لزوم اجرای برنامه‌های آموزشی با محوریت خانواده در مراکز بهداشتی درمانی، بیمارستان‌ها و رسانه‌های گروهی است.

Title: بررسی میزان آلودگی فلزات سنگین (مس، سرب، روی، کادمیوم، آهن و منگنز) در منابع تأمین‌کننده آب شرب شهر نورآباد لرستان در سال 1392

http://yafte.lums.ac.ir/article-1-2293-fa.html

مقدمه: آب سالم در طول مسیر از منابع تأمین تا محل مصرف مراحلی را طی می‌کند که در عبور از این مراحل ممکن است دچار بعضی از موارد آلودگی از قبیل آلودگی عناصر فلزی سنگین گردد. لذا هدف از این تحقیق بررسی میزان آلودگی فلزات سنگین (مس، سرب، روی، کادمیوم، آهن و منگنز) در منابع تأمین‌کننده آب شرب شهر نورآباد لرستان در سال 1392 می‌باشد. مواد و روش ­ها: در این مطالعه که از نوع توصیفی– تحلیلی ‌مقطعی می‌باشد طی 6 ماه از 7 حلقه چاه منابع تأمین آب شرب و 2 مخزن تأمین آب شهر نورآباد نمونه برداری به روش نمونه‌برداری طبقه‌ای انجام گردید. بدین این منظور پارامترهای فلزاتی نظیر مس، سرب، روی، کادمیوم، آهن و منگنز توسط دستگاه پلاروگراف و همچنین هدایت الکتریکی، سولفات، کلراید و کل جامدات محلول مطابق استاندارد متد اندازه‌گیری شد. یافته­ ها: نتایج نشان داد که غلظت فلزات مورد بررسی در منابع تأمین آب پایین‌تر از استاندارد‌های ملی و سازمان جهانی بهداشت بوده و در شبکه توزیع بعضی از فلزات بیشتر از استاندارد ارزیابی گردید. همچنین با توجه به نتایج غلظت فلزات مورد بررسی در فصل زمستان بیشتر از فصل پاییز بدست آمد. بحث و نتیجه ­گیری: به‌طور کلی غلظت فلزات سنگین مورد بررسی در منابع تأمین آب شهر‌نورآباد زیر حد استاندارد ملی و سازمان جهانی بهداشت بوده و مشکلی برای مصرف‌کنندگان آب وجود ندارد؛ اما با این وجود به دلیل بهداشت عمومی و بالاتر بودن این فلزات در شبکه توزیع بایستی پایش‌های مستمر و منظم بر روی غلظت فلزات‌سنگین آب شرب منطقه از طرف سازمان‌های مسئول صورت گیرد.

Title: کرم واژینال کلیندامایسین در پیشگیری از پره‌ترم لیبر

http://yafte.lums.ac.ir/article-1-2294-fa.html

مقدمه: زایمان زودرس یکی از شایع‌ترین علل مرگ میر پری‌ناتال در سراسر جهان است. هدف این مطالعه تعیین تأثیر کرم واژینال کلیندامایسین در پیشگیری از زایمان زودرس بود. مواد و روش ­ها: مطالعه حاضر یک کار آزمایی بالینی است که بر روی 236 نفر از زنان باردار مراجعه کننده به مطب خصوصی همکار پژوهشی که سن حاملگی بین 20-13 هفته داشتند انجام شد. نمونه اسمیر واژینال تهیه و درصورتی‌که نتیجه غیرطبیعی بود فرد به‌طور تصادفی در یکی از دو گروه شاهد یا مورد قرار می‌گرفت. گروه مورد تحت درمان با کرم واژینال کلیندامایسین به مدت سه شب نوبت اول و هفت شب نوبت دوم قرار می‌گرفتند و جهت گروه شاهد مطابق روتین درمانی صورت نمی‌گرفت. اطلاعات با استفاده از پرسشنامه گرد آوری و مورد تجزیه و تحلیل قرار گرفت. یافته ­ها: میزان بروز زایمان زودرس در گروه شاهد بیشتر از گروه تحت درمان با کلیندامایسین (12% در مقایسه با 7%) ولی این تفاوت در حدی نبود که از نظر آماری معنی دار گردد. علاوه بر بروز بیشتر زایمان زودرس در گروه شاهد سن ختم بارداری نیز پایین‌تر و مرگ و میر پری‌ناتال نیز در گروه شاهد به‌طور معنی داری بیشتر از گروه مورد بود. بحث و نتیجه ­گیری: باوجود اینکه تفاوت معنی داری در میزان بروز پره‌ترم لیبر بین دو گروه مشاهده نشد ولی میزان بروز پره‌ترم لیبر در گروه شاهد بیشتر، سن بارداری کمتر و میزان مرگ و میر بیشتر بود. به نظر می‌رسد کلیندامایسین می‌تواند در کاهش مرگ میر پری‌ناتال ناشی از زایمان زودرس مؤثر باشد.

Title: بررسی شیوع سرمی آلودگی به سرووارهای مختلف لپتوسپیرا در میان شالیکاران ویسیان استان لرستان

http://yafte.lums.ac.ir/article-1-2295-fa.html

مقدمه: لپتوسپیرا متعلق به راسته اسپیروکت­ها است که موجب بیماری لپتوسپیروز می‌شود. لپتوسپیروز یک بیماری عفونی مشترک میان انسان و دام با علائم بالینی بسیار متغیر است. هدف مطالعه تعیین شیوع سرمی سرووارهای مختلف لپتوسپیرا در میان شالیکاران ویسیان در استان لرستان بوده است. مواد و روش ­ها: 200 شالیکار ویسیان (مرد و زن) به‌صورت تصادفی در بهار 1393 نمونه‌گیری (نمونه خون) شدند. نمونه‌های خون به آزمایشگاه منتقل و سرم آن‌ها جدا شد، سرم‌ها تا زمان انجام تست میکروآگلوتیناسیون میکروسکوپی  (MAT)در فریزر 20- درجه سانتی‌گراد نگهداری شدند. نمونه‌های سرمی به روش MAT مورد بررسی قرار گرفتند. تیتر نهایی سرم‌ها با رقت سازی 1:200، 1:400 و 1:800 تعیین گردید. یافته ­ها: از مجموع 200 نمونه سرمی مورد آزمایش، 60 نمونه (30%) عیار مثبت سرمی 1:100 (1:100≤) را نشان دادند. از میان 60 نمونه سرمی مثبت، 38 نمونه (33/63%) با سرووار گریپوتیفوزا و 22 نمونه (67/36%) با سرووار کانیکولا واکنش مثبت نشان دادند. میزان عفونت در مردان 65% و در زنان 35% بود. از نظر آماری میان جنسیت و نتایج حاصل از آزمایش MAT ارتباط معنی داری (02/0p=) وجود دارد. بیشترین و کمترین موارد مثبت به ترتیب در گروه سنی بالای 50 سال و 30-21 سال قرار داشتند. بحث و نتیجه ­گیری: لپتوسپیروز در میان شالیکاران یک بیماری شایع است و در منطقه ویسیان با توجه به شالیکاری به روش سنتی به‌عنوان یک بیماری عفونی مهم مطرح می‌گردد؛ لذا برای کنترل و پیشگیری از این بیماری باید اقدامات بهداشتی و ایمنی گسترش یابد. همچنین با بکارگیری روش‌های مکانیزه کشاورزی تا حدود زیادی از شیوع لپتوسپیروز در این منطقه جلوگیری می‌شود.

Title: بررسی و شناسایی ژنهای کروم سنسینگ در سویه های سودوموناس آئروژینوزا جداشده از نمونه های بالینی انسانی به روش Multiplex PCR و تعیین مقاومت آنتی بیوتیکی

http://yafte.lums.ac.ir/article-1-2296-fa.html

مقدمه: سودوموناس آئروژینوزا یک پاتوژن فرصت طلب و عامل 10 الی 15 درصد از عفونت­های بیمارستانی می‌باشد. وجود ژن‌های حدت یکی از مهم‌ترین مکانیسم‌های تهاجمی سودوموناس آئروژینوزا می‌باشد و این موضوع از نظر پزشکی از اهمیت ویژه­ای برخوردار است. بیان بسیاری از ژن‌های بیماریزای باکتری سودوموناس آئروژینوزا به‌وسیله یک سیستم ژنی به نام سیستم Quorum Sensing (QS) کنترل و تنظیم می­گردند. کروم سنسینگ سیستم ارتباطی سلول به سلول با استفاده از مولکول‌های کوچک SMs در ارگانیسم‌های تک سلولی است. هدف از تحقیق بررسی شیوع ژن‌های دخیل در سیستم کروم سنسینگ در سویه‌های سودوموناس آئروژینوزا جداشده از منابع انسانی می‌باشد. مواد و روش ­ها: در این مطالعه، 60 نمونه سودوموناس آئروژینوزا از نمونه‌های گوارش انسانی تهیه و در محیط‌های اختصاصی کشت و توسط تست‌های تشخیصی و افتراقی تأیید گردیدند. آزمون Multiplex PCR برای ردیابی ژن‌های مورد نظر انجام گرفت. آزمون حساسیت آنتی بیوتیکی علیه 10 عامل ضد میکروبی صورت گرفت. یافته ­ها: نتایج Multiplex PCR نشان داد که میزان فراوانی ژن‌های rhlR  5 درصد، ژنlasR  3/48 درصد و ژنlasI  60 درصد بوده، در صورتی که ژن‌های lasB،apr ، rhlAB و rhlI در هیچ یک از نمونه‌ها شناسایی نگردیدند. در آزمون حساسیت آنتی بیوتیکی، بیشترین میزان مقاومت نسبت به آنتی بیوتیک‌های آمیکاسین، آموکسی‌سیلین و سفوتاکسیم و بیشترین میزان حساسیت به آنتی بیوتیک‌های سیپروفلوکساسین و سفتازیدیم گزارش گردیده است. بحث و نتیجه­ گیری: ژن‌های سیستم QS فراوانی بالایی در بین سویه‌های سودوموناس آئروژینوزا جداشده از منابع انسانی دارند.

Title: بررسی اثر پروتئین core ویروس هپاتیت HCV) C) بر روی بیان miR-150

http://yafte.lums.ac.ir/article-1-2297-fa.html

مقدمه: ویروس هپاتیت C (HCV) به عنوان یکی از عوامل اصلی بیماری زا برای بیماری‌های مزمن کبدی محسوب می‌شود. مطالعات قبلی نشان داده‌اند که پروتئین‌های ویروس HCV می‌توانند با شبکه های تنظیمی ژن از قبیل microRNAs میانکنش برقرار کنند. هدف از این مقاله بررسی اثر پروتئین core ویروس HCV بر روی بیان miR-150 در یک مدل کشت سلولی بود. مواد و روش ­ها: پلاسمیدهای بیان کننده طول کامل پروتئین Core ویروس HCV به داخل رده سلولی Huh7 ترانسفکت گردیدند، در حالیکه در کنار آن از یک پلاسمید بیان کننده GFP خالی به عنوان یک گروه کنترل منفی استفاده شد. سپس RNA تام استخراج شده و Real-Time PCR جهت اندازه گیری سطح بیان miR-150 انجام شد. علاوه براین، تست trypan blue exclusion جهت بررسی اثر پروتئین core بر روی زنده‌مانی سلولی نیز انجام گرفت. یافته ­ها: آنالیز بیان ژن miR-150 در سلول‌های Huh7 نشان داد که پروتئین core درون زاد ویروس HCV می‌تواند به‌طور معناداری باعث کاهش بیان miR-150 در قیاس با پلاسمید کنترل بیان کننده GFP و سلول طبیعی شود (01/0P<). در کنار این، پروتئین core هیچگونه اثرات کشندگی سلولی یا تحریک کننده‌ای را بر روی سلول‌های کبدی پس از انجام تست trypan blue exclusion نشان نداد (05/0P<). بحث و نتیجه­ گیری: مطالعه ما نشان می‌دهد که پروتئین core ویروس HCV می‌تواند منجر به کاهش بیان miR-150 شود. این نتایج بیانگر آنست که میانکنش پروتئین‌های HCV با ماشین تنظیمی سلولی ممکن است در پاتوژنز مزمن بیماری‌های کبدی نقش داشته باشد.

Title: بررسی نقش نارسایی شناختی و هیجانی در پیش بینی عضویت در گروه های معتاد و غیرمعتاد

http://yafte.lums.ac.ir/article-1-2298-fa.html

مقدمه: پژوهش حاضر با هدف بررسی نقش نارسایی شناختی و هیجانی در پیش­بینی عضویت در گروه­ های معتاد و غیرمعتاد انجام شد. مواد و روش ­ها: این پژوهش یک پژوهش بنیادی و از نظر نوع بررسی پس­رویدادی (مقطعی- مقایسه­ای) است که به‌صورت گذشته­نگر انجام شد. جامعه آماری پژوهش حاضر را مردان سوء­مصرف­کننده مواد مراجعه کننده به مراکز ترک اعتیاد یکی از شهرستان‌های استان لرستان در سال 1393 تشکیل می­دادند. نمونه انتخابی از این جامعه شامل 100 نفر از افراد سوءمصرف­کننده مواد و 100 نفر از افراد عادی (از میان همراهان بیماران) بودند که به روش نمونه ­گیری هدفمند انتخاب شدند. همه شرکت­ کنندگان پرسشنامه­ های نارسایی شناختی (CFQ) و نارسایی هیجانی (TSA-20) را تکمیل نمودند. جهت تجزیه و تحلیل داده­ ها از روش تحلیل ممیز استفاده شد. آنالیز با استفاده از برنامه آماری SPSS-20 صورت گرفت. یافته­ ها: در تحلیل ممیز به روش همزمان، هفت متغیر پیش­بین (دشواری در توصیف و شناسایی احساسات، تفکر معطوف به بیرون، مشکلات حافظه، حواس­پرتی، اشتباهات سهوی و عدم یادآوری اسامی) و در روش گام به گام سه متغیر پیش­بین (نارسایی شناختی، دشواری در توصیف احساسات و مشکلات مربوط به حافظه) برای تنها تابع ممیز از اختلاف بین دو گروه معتاد و غیرمعتاد تبیین می ­شود. بحث و نتیجه ­گیری: نتایج نشان داد که افراد معتاد و غیرمعتاد را می­توان از روی نارسایی شناختی و هیجانی تمییز داد.

Title: تأثیر نانهای مصرفی در ایران بر سطح قند خون بیماران مبتلا به دیابت نوع 2

http://yafte.lums.ac.ir/article-1-2299-fa.html

مقدمه: افزایش سطح قند خون پس از صرف غذا، شایع‌ترین اختلال در افراد دیابتی است. از آنجائی که نان یکی از منابع اصلی غذایی در ایران است، هدف از این مطالعه بررسی تأثیر پنج نوع نان مصرفی در ایران (باگت، لواش، تافتون، سنگک و نان جو) بر سطح قند خون بیماران مبتلا به دیابت نوع 2 و افراد سالم بود. مواد و روش­ ها: در این مطالعه 150 شرکت کننده در دو گروه مساوی بیماران دیابتی نوع 2 و افراد سالم مورد مطالعه قرار گرفتند. هر دو گروه به‌صورت تصادفی به پنج زیرگروه مساوی تقسیم شده، سپس در هر گروه یکی از 5 نوع نان مورد آزمایش که حاوی 50 گرم کربوهیدرات در دسترس بود، داده شد. میزان قند خون در شروع مطالعه، 60 و 120 دقیقه پس از مصرف نان‌ها، اندازه‌گیری شد. یافته­ ها: نتایج نشان داد در هر دو گروه، بالاترین افزایش گلوکز خون بعد از مصرف نان باگت مشاهده شد. نوسانات قند خون پس از 120 دقیقه، در آزمودنی‌هایی که نان سنگک و جو مصرف کرده بودند، به‌طور معنی داری کمتر از مصرف کنندگان نان باگت، لواش و تافتون بود (01/0P<). بحث و نتیجه ­گیری: نان‌های جو و سنگک تأثیر بیشتری بر کنترل گلوکز خون آزمودنی‌ها داشتند که می‌تواند نقش مهمی در تنظیم قند خون بیماران دیابتی ایفا می‌کند.

Title: ارزیابی درونی گروه فیزیولوژی دانشگاه علوم پزشکی لرستان

http://yafte.lums.ac.ir/article-1-2300-fa.html

مقدمه: با توجه به نقش نظام آموزشی، لازم است که در طراحی و اجرای فعالیت‌های آن از مطلوب‌ترین شیوه ­ها استفاده شود. ارزیابی درونی فرایندی است که در آن اعضاء گروه آموزشی، هدف‌های گروه را تصریح کرده و عملکرد آن را مورد قضاوت قرار داده و سپس نقش خود را بازنگری و برای عملکرد بهتر، گام‌های اساسی بر می‌دارند. هدف از انجام این مطالعه، ارزیابی درونی گروه فیزیولوژی دانشگاه علوم پزشکی لرستان می ­باشد. مواد و روش­ها: این ارزیابی یک مطالعه توصیفی- مقطعی بوده که در سال تحصیلی 94-93 اجرا شد. جمع‌آوری اطلاعات بر اساس پرسش‌نامه، مشاهده و مصاحبه انجام و ارزیابی در 6 حوزه مختلف صورت گرفت. داده­ها با استفاده از مقیاس اندازه گیری لیکرت با دامنه امتیاز 0 تا 5 در رتبه ­های مطلوب، نسبتاً مطلوب و نامطلوب طبقه ­بندی گردیدند. یافته ­ها: حوزه مدیریت گروه (با میانگین اکتسابی 23/4) اعضای هیأت علمی (57/3)، دوره ­های آموزشی (96/3)، برنامه­ های درسی دپارتمان فیزیولوژی (75/3) رتبه مطلوب و حوزه ­های امکانات و تجهیزات آموزشی و پژوهشی (92/2) وضعیت پژوهشی اعضای هیأت علمی (73/2) رتبه نسبتاً مطلوب را دارا بودند. بحث و نتیجه ­گیری: گروه فیزیولوژی دانشگاه علوم پزشکی لرستان در مجموع حوزه‌های مورد ارزیابی با میانگین 39/3 طبق مقیاس لیکرت رتبه مطلوب را داراست.

Title: بررسی میزان آلودگی به انگل توکسوپلاسما گوندی با روش‌های پارازیتولوژیک و سرولوژیک در گربه‌های ولگرد شهر خرم‌آباد، سال 1393

http://yafte.lums.ac.ir/article-1-2302-fa.html

مقدمه: توکسوپلاسما از شایع‌ترین انگل­های مشترک انسان و حیوان است. گربه­ های ولگرد به علت دسترسی به مواد گوشتی و شکار جونده و پرنده آلوده شده و با پخش کیست، نقش مهمی در آلوده سازی میزبان­ های واسط ایفا می‌کنند. هدف این مطالعه جستجوی آنتی­بادی ضد توکسوپلاسما در خون و اواوسیست در مدفوع، ارتباط برخی متغیرها مثل سن و جنس و منطقه جغرافیایی زندگی گربه ­ها با میزان آلودگی آن‌ها بود. مواد و روش ­ها: از 125 قلاده گربه ولگرد خرم آباد پس از صید با تله زنده گیر و بیهوشی و ثبت مشخصات آن‌ها، سرم خون آن‌ها تهیه و به روشIgG- ELISA  آزمایش شد. جهت بررسی دفع اواوسیست، مدفوع گربه­ها از طریق نمونه برداری رکتال تهیه و با روش فلوتاسیون شیتر بررسی گردید. جهت تعیین ارتباط متغیرها، سطح معنی‌داری P کمتر از 05.0 در نظر گرفته شد. یافته ­ها: 80 گربه (64%) دارای IgG ضد توکسوپلاسما، 42 مورد منفی (6/33%) و 3 مورد (4/2%) حدمرزی بودند. بین جنس و منطقه جغرافیایی صید گربه­ها با مثبت بودن سرمی ارتباط معنی دار وجود نداشت. بین سن و مثبت بودن سرمی IgG ارتباط معنی دار مشاهده شد (021.0P<). همچنین هیچ‌گونه اواوسیستی که از نظر اندازه شبیه توکسوپلاسما باشد در مدفوع دیده نشد. در چند نمونه، اواوسیست مشابه توکسوپلاسما با اندازه بزرگ­تر مشاهده و ایزوسپورا تشخیص داده شد. بحث و نتیجه ­گیری: مطالعه حاضر نشان داد آلودگی به توکسوپلاسما در گربه ­های منطقه نسبتاً بالاست. در صورت عدم رعایت اصول بهداشتی احتمال آلودگی انسان قابل توجه می‌باشد.

Title: مقایسه نتایج بیوپسی روده باریک و تست سرولوژیک آنتی ترانس گلوتامیناز بافتی در کودکان مبتلا به بیماری سلیاک در شهر اهواز در سال 94

http://yafte.lums.ac.ir/article-1-2304-fa.html

مقدمه: سلیاک یک بیماری خودایمنی می­باشد که به دنبال مصرف غذای حاوی گلوتن به ویژه در افرادی که استعداد ژنتیکی دارند، رخ می دهد. هدف از این مطالعه مقایسه روش های موجود برای تشخیص بیماری سلیاک و ارتباط سنجی میان این روش ها می ­باشد. مواد و روش ­ها: در این مطالعه، 50 بیمار با شک بالینی سلیاک وارد مطالعه شدند. پس از انجام آزمایش IgA anti-TTG به روش الایزا، بیماران تحت عمل آندوسکوپی قرار گرفته و تقسیم بندی بافت شناسی بر اساس نوع مارش انجام شد و نتایج با تست IgA anti-TTG مقایسه گردید. یافته­ ها: 50 بیمار شامل 23 پسر و 27 دختر با میانگین سنی 8.36 سال بودند. آزمایشات سرولوژیک در 45 نفر مثبت و در 5 نفر منفی بود. در بررسی هیستوپاتولوژیک بیماران، طرح غیر طبیعی در تمام موارد مشاهده گردید که اکثرا در گروه مارش III قرار گرفتند. بین نتایج سرولوژیک و یافته های بیوپسی بیماران ارتباط معنی داری به دست آمد (014/0 p-valu=). در حالی که میان سن بیماران و نتایج بیوپسی ارتباط معنی داری دیده  نشد. بحث و نتیجه ­گیری: بر اساس نتایج به دست آمده در این مطالعه بین یافته های هیستوپاتولوژیک و یافته های سرولوژیک رابطه معنی داری مشاهده گردید، رویکرد تشخیصی مطرح شده در این مطالعه می تواند در ارزیابی هر چه بهتر بیماری سلیاک مفید واقع شود. در پایان، برای تشخیص قطعی بیماری سلیاک انجام آزمایشات مولکولی و تعیین هاپلوتایپ های HLADQ نیز پیشنهاد می گردد.

Title: مقایسه فعالیت‌ الکترومیوگرافی (EMG) عضلات درگیر در ‌مرحله رهایی پرتاب نیزه قهرمانان معلول مرد جهان و پارالمپیک

http://yafte.lums.ac.ir/article-1-2305-fa.html

مقدمه: هدف از این مطالعه شناسایی متغیر الگوی فعالیت عضلانی (آمپلی تود) عضلات پکتورالیس ماژور، بخش قدامی دلتویید و تری‌سپس پرتاب‌گران نیزه قهرمان جهان و پارالمپیک مرد کشور با استفاده ازEMG  بود. مواد و روش­ ها: فعالیت الکتریکی عضلات پکتورالیس ماژور، بخش قدامی دلتویید و تری‌سپس 24 نفر پرتابگر نیزه معلول نخبه مرد ایرانی کلاس‌های مختلف نشسته و ایستاده با استفاده از دستگاه الکترومیوگرافی سطحی انجام شد. در این مطالعه از تحلیل واریانس یک طرفه (One way ANOVA) و آزمون تی(t) مستقل در سطح معنی‌داری 05/0 استفاده گردید. یافته­ ها: بین الگوی فعالیت عضلانی‌(آمپلی‌تود) عضلات پکتورالیس ماژور و دلتویید قدامی کلاس‌های نشسته، عضله تری‌سپس  کلاس‌های ایستاده و عضله پکتورالیس ماژور کلاس‌های نشسته با ایستاده قهرمانان معلول در طی رهایی نیزه تفاوت معنی‌داری وجود داشت (001/0>p). در حالی‌که در آمپلی‌تود  عضله تری‌سپس کلاس‌های نشسته پکتورالیس ماژور و بخش قدامی عضله دلتویید کلاس­ های ایستاده و بخش قدامی عضله دلتویید و تری­سپس کلاس­ های نشسته و ایستاده تفاوت معنی‌داری مشاهده نگردید. بحث و نتیجه­ گیری: به طور متوسط میانگین تمامی متغیرهای مورد نظر در کلاس‌های نشسته بیشتر از کلاس ­های ایستاده بود. بالاترین مقادیر آمپلی‌تود  به ترتیب متعلق به بخش قدامی عضله دلتویید، پکتورالیس ماژور و تری‌سپس گروه ­های نشسته بود. این امر نشان دهنده استفاده بیشتر از عضلات مورد نظر بواسطه اختلال و معلولیت و عدم کارآیی عضلات پایین تنه پرتاب‌گران نشسته نسبت به پرتاب‌گران ایستاده می­باشد. مقایسه آمپلی­تود فعالیت الکترومیوگرافی عضلات مورد نظر در همه کلاس ­ها و معلولیت ­ها نشان داد که آنها نقش مهمی در عملکرد  پرتاب‌گران معلول ایفا می کنند.
